# Water body extraction from high spatial resolution remote sensing images based on enhanced U-Net and multi-scale information fusion

**DOI:** 10.1038/s41598-024-67113-7

**Published:** 2024-07-12

**Authors:** Huidong Cao, Yanbing Tian, Yanli Liu, Ruihua Wang

**Affiliations:** College of Information and Control Engineering, Qingdao University of Technology, Qingdao, 266525 China

**Keywords:** Remote sensing, Water body extraction, Deep learning technique, U-net, Environmental sciences, Hydrology

## Abstract

Employing deep learning techniques for the semantic segmentation of remote sensing images has emerged as a prevalent approach for acquiring information about water bodies. Yet, current models frequently fall short in accurately extracting water bodies from high-resolution remote sensing images, as these images often present intricate details of terrestrial objects and complex backgrounds. Vegetation, shadows, and other objects close to water boundaries have increased similarity to water bodies. Moreover, water bodies in high-resolution images have different boundary complexities, shapes, and sizes. This situation makes it somewhat challenging to accurately distinguish water bodies in high-resolution images. To overcome these difficulties, this paper presents a novel network model named EU-Net, specifically designed to extract water bodies from high-resolution remote sensing images. The proposed EU-Net model, with U-net as the backbone network, incorporates improved residual connections and attention mechanisms, and designs multi-scale dilated convolution and multi-scale feature fusion modules to enhance water body extraction performance in complex scenarios. Specifically, in the proposed model, improved residual connections are introduced to enable the learning of more complex features; the attention mechanism is employed to improve the model's discriminative ability by focusing on important channels and spatial areas. The implemented multi-scale dilated convolution technique enhances the model's receptive field while maintaining the same number of parameters. The designed multi-scale feature fusion module is capable of processing both small-scale details and large-scale structures in images, while simultaneously modeling the spatial context relationships of features at different scales. Experimental results validate the superior performance of EU-Net in accurately identifying water bodies from high-resolution remote sensing images, outperforming current models in terms of water extraction accuracy.

## Introduction

Remote sensing imagery effectively captures terrestrial information, with optical remote sensing images often containing rich attribute information of terrestrial objects across multiple spectral bands, ranging from visible light to far-infrared^1–5^. In the visible spectrum, the combination of red, green, and blue can generate natural color images similar to what the human eye perceives, which is especially important for analyzing surface vegetation coverage, water bodies, and man-made structures^6–11^. Initially, McFeeters proposed the Normalized Difference Water Index (NDWI)^12^, inspired by the Normalized Difference Vegetation Index (NDVI)^13^, utilizing the difference in spectral band reflectance to identify specific features. This approach has had a profound impact on traditional non-machine learning waterbody extraction research. However, despite significant progress in improving the accuracy of waterbody recognition, these methods still face some limitations and challenges: determining the threshold for water indices such as MNDWI and AWEI to differentiate between water and non-water areas presents notable complexity, as the spectral characteristics of water bodies can vary significantly across different geographical locations and environmental conditions. Environmental changes, such as seasonal variations and different weather conditions, can also affect the applicability of these thresholds, requiring adaptive adjustments. Moreover, even with the introduction of the SWIR band to suppress non-water features, distinguishing water bodies from other land features in complex surface coverage conditions, such as densely vegetated areas or urban landscapes, remains challenging. Especially without proper threshold adjustments to match specific monitoring scenarios, this land feature confusion can lead to misclassification, thereby affecting the accuracy of waterbody detection^14^.

In machine learning, particularly in image classification tasks, feature extraction is a critical process. It involves identifying and extracting information from raw image data. In the past, feature extraction techniques in the early stages heavily relied on manually crafted approaches such as scale-invariant feature transformation (SIFT)^15^ and histogram of oriented gradients (HOG)^16^, which were tailored explicitly for distinct image feature types. The extracted features are subsequently utilized as inputs for classifiers, such as Random Forest (RF)^17^, to perform surface object classification. However, these handcrafted features are task-specific, making feature extraction from an extensive array of diverse datasets impractical. Lately, algorithms based on deep learning have consistently topped the charts in achieving the greatest accuracy across a range of visual recognition tasks^18^. Deep learning, a subfield of machine learning, employs algorithms inspired by neural networks to simulate the cognitive processes of the human brain in information processing. This approach allows for learning and decision-making from large datasets. Yu utilized Convolutional Neural Networks (CNNs) for the extraction of water bodies from imagery captured by Landsat satellites^19^. Yang incorporated adaptive pooling techniques into Convolutional Neural Networks (CNNs) as part of his research to efficiently detect urban water bodies in high-resolution Chinese remote sensing data. This was accomplished explicitly by leveraging ZY-3 and GF-2 multispectral images^20^. Li merged the capabilities of deep learning and Google Earth Engine for urban water extraction. The integration involved the amalgamation of Google Earth Engine (GEE) with a multi-scale Convolutional Neural Network (MSCNN), ensuring precise identification of urban water without relying on subjective judgments^21^. Zhou et al. developed UNet++ through a nested U-structure^22^, and Md et al. constructed R2U-Net by introducing RNN^23^. Fully Convolutional Networks (FCNs)^24^ were the first CNNs designed end-to-end for pixel-level tasks. Abstract features are extracted from input images using FCNs, and labels are assigned to individual pixels within the feature maps generated by the final convolutional layer. Nevertheless, FCNs often encounter challenges in preserving the acquired knowledge from the initial or shallow convolutional layers, which capture low-level characteristics. In recent years, to enhance the semantic segmentation performance of CNNs in computer vision, further developments and improvements in network structures have continued, such as U-net^25^ and DeeplabV3+^26^. The emergence of Transformer^27^ offers a new perspective on improving object detection. Transformers replace traditional recurrent neural networks (RNNs) and convolutional neural networks (CNNs) entirely based on attention mechanisms, enabling more efficient and flexible sequence-to-sequence (seq2seq) modeling. Vision Transformer (ViT)^28^ is established by Dosovitskiy et al. in 2020, applying the Transformer architecture to image classification tasks. ViT divides the image into a series of fixed-size image patches, and then inputs these patches into a standard Transformer for processing, achieving image classification. This method demonstrates that the Transformer architecture can also perform well in image processing tasks when trained on a large number of GPUs.In recent years, numerous architectures combining CNN and Transformer have emerged, aiming to leverage the powerful feature extraction capabilities of CNNs and the long-distance dependency modeling of Transformers, thus creating more robust models. Chen et al. combined CNN and Transformer to create a network specifically for lake extraction^29^. Zhang et al. improved the accuracy of CNN-Transformer architecture in remote sensing image waterbody extraction by reducing data noise^30^. Kang et al. utilized a dual-stream CNN combined with Transformer to create a CNN-Transformer network specifically for lake extraction^31^. While the combination of CNN and Transformer can significantly improve the accuracy of water body segmentation, the Transformer's handling of long-range dependencies requires substantial computational resources^32^. CNN-based water body segmentation, while maintaining accuracy, offers higher computational efficiency and broader application potential. Therefore, research into CNN based approaches remains meaningful.

This study proposes an enhanced lightweight network model, EU-Net. Although the latest UNeXt^33^ model has shown significant performance improvements over the U-net model in specific tasks, its complex structure may limit its generalization ability across different types of data or tasks. Therefore, this paper proposes a model that uses U-net as the backbone. EU-Net is designed for waterbody remote sensing images with complex features and textures, including the introduction of spatial and channel attention mechanisms to improve classification accuracy and incorporating improved residual connections to enhance model generalization. In the EU-Net encoder-decoder architecture, a multi-scale dilated convolution module is cleverly designed. This module uses three different dilation rates in the down-sampling and up-sampling layers, effectively increasing the network's receptive field. At the middle of the model, a multi-scale feature fusion module is meticulously designed. This innovative approach is particularly important in handling complex visual tasks.EU-Net significantly enhances its ability to recognize objects of different types, sizes, and shapes by extracting unique information at different scales and utilizing this multi-scale feature fusion. This method not only enhances the robustness of the model but also improves the model’s classification accuracy. In comparative experiments, this paper focuses on comparing recent lightweight networks, and the results show that this network outperforms other methods.

## Methods

### The overall structure of the EU-Net model

The proposed EU-Net network model and its innovative features will be detailed in this section. The key to water body extraction is to obtain features with high discrimination for water bodies from images. To address the limitations associated with employing a convolutional neural network for training and prediction, exploring alternative approaches that utilize an image block encompassing the individual pixel is crucial when classifying it.

Firstly, there is a significant storage overhead, as the convolutional kernels continuously slide over windows for each pixel, with each sliding window being classified by the convolutional neural network. A small field of view can lead to insufficient capture of local information, while a large field of view may result in the loss of edge information. Secondly, when sliding the window, the adjacent pixel blocks are largely repetitive, leading to inefficient computations as each pixel block is convolved individually. Lastly, the pixel block's size limits the receptive field's size.

**Figure 1:**
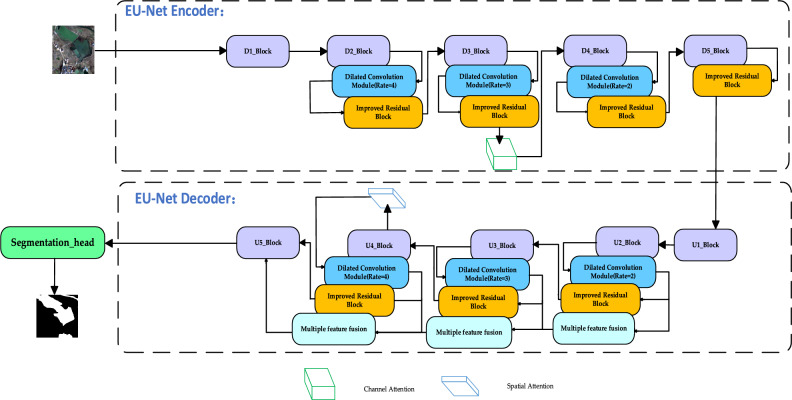
EU-Net structure diagram.

Based on these challenges, the EU-Net network structure has been designed, as illustrated in Fig. 1. The network comprises two main parts: the upper part for down-sampling operations and the lower part for up-sampling operations.

In the upper part of EU-Net, each module (D1_Block to D5_Block) consistently involves two 3 × 3 convolutions followed by a ReLU activation function and a 2 × 2 max-pooling layer for downsampling. With each downsampling iteration, the number of feature channels gradually doubles until reaching the lowest resolution. Multi-scale dilated convolutions are inserted into the middle three layers of the downsampling process (D2_Block to D4_Block). Dilated convolutions, by introducing gaps (dilation factors) between convolution kernel elements, can expand the receptive field without increasing the number of parameters. This allows the model to capture a wider range of contextual information, facilitating the processing of global features. Additionally, a channel attention mechanism is implemented in the middle part of the D3_Block. The channel attention mechanism, by assigning different weights to different channels, can emphasize important feature channels and suppress less important ones. This allows the model to better focus on features crucial for the current task (e.g., classification, detection, segmentation), thereby improving model performance.

In the lower part of EU-Net, each module (U1_Block to U5_Block) retains a large number of feature channels during the upsampling process, allowing the network to transfer contextual information to higher resolution layers. Each layer in this region includes a 2 × 2 transposed convolution operation for upsampling, effectively halving the number of feature channels. It also involves the fusion of feature maps from the corresponding downsampling layer, followed by two 3 × 3 convolutions and ReLU activations. Multi-scale dilated convolution modules and multi-scale feature fusion modules are included from U2_Block to U4_Block. Different scale feature maps can capture targets of different sizes; small-scale feature maps are suitable for capturing large targets, while large-scale feature maps are suitable for capturing small targets. By incorporating multi-scale feature fusion modules, the model can simultaneously handle targets of various sizes, improving detection and segmentation accuracy. A spatial attention mechanism is implemented in the middle part of the U4_Block. The spatial attention mechanism, by assigning different weights to different positions in the image, can emphasize crucial regions in the image. This allows the model to better focus on key areas in the image, enhancing task performance. Finally, the network is connected to a segmentation head for classification.

EU-Net integrates an improved residual block throughout its architecture. This residual block adjusts feature map values by incorporating a Sigmoid activation function into the residual connection. The Sigmoid function maps inputs to the range (0, 1), effectively controlling information flow by enhancing significant features and suppressing irrelevant ones. The improved residual connection maintains the basic characteristics of a residual block, namely enhancing gradient flow through skip connections, while simultaneously adjusting the input for more stable and effective gradient propagation.

### Residual connections for feature enhancement

In remote sensing, especially when extracting water bodies, it's crucial to capture intricate details like small water bodies. The original U-net architecture, despite its successes, exhibited limitations when applied to complex aquatic environments. GID-Water and WHDLD dataset's complexity, characterized by numerous small water bodies interspersed with various land cover types, posed a significant challenge. The inherent downsampling process in U-net, while effective for capturing broader contextual information, resulted in a substantial loss of these crucial fine details. As a result, the model's ability to delineate small and intricate water bodies was compromised, leading to reduced accuracy and precision in the water body extraction task.

To address this issue, this study proposes the integration of residual connections into the U-net architecture, a modification aimed at preserving the high-resolution features essential for detecting small water bodies. Residual connections, or skip connections, are a fundamental component of the ResNet^34^ architecture, which has seen tremendous success in various image recognition tasks. These connections allow the direct flow of information across layers, effectively creating shortcuts in the network. Through this approach, they address the issue of data loss during the downsampling stage and maintain the essential finer details needed for precise delineation of water bodies.

Incorporating residual connections also tackles the problem of vanishing gradients. This issue often arises in deep neural networks where, with increasing network depth, the gradients essential for training become extremely small, leading to a stagnation in the network's learning process. By providing alternate pathways for the gradient flow, residual connections ensure a more robust and stable training process, allowing the construction of deeper networks capable of capturing more complex features without the risk of training stagnation.

As shown in Fig. 1, in the EU-Net model with residual connections, each convolutional block is supplemented with a shortcut that bypasses one or more layers. In the forward pass, the output of a block is combined with its input, and this occurs prior to the activation step. This process allows the network to learn modifications to the identity mapping rather than the entire transformation, which has been shown to be easier and more effective for deep networks. Consequently, the network's focus is on acquiring knowledge about the residual mappings that enhance the characteristics, thereby enhancing the model's capacity to comprehend the intricate details of minor water bodies. Experiments show that introducing residual connections into the U-net architecture results in significant improvements in terms of accuracy metrics. The improvements are particularly pronounced in challenging scenarios where traditional U-net would struggle, such as areas with dense vegetation near water bodies, narrow streams, or water bodies with irregular shapes. The residual U-Net's efficacy in preserving and enhancing intricate details throughout the network renders it a dependable solution for water body extraction, particularly in high-resolution remote sensing imagery where each pixel holds significant value.

**Figure 2:**
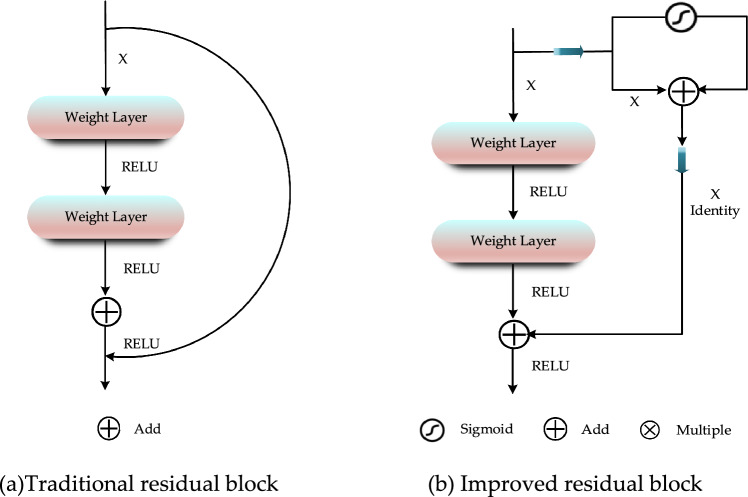
Residual block.

We have made improvements in the details of the residual connections. Figure 2 displays the original residual connections and the improved residual connections. As illustrated in Fig. 2b, we introduce a gating mechanism controlled by the Sigmoid activation function in the skip connection of the residual block. This mechanism regulates the information flow through the skip connection by element-wise multiplying the direct path of the skip connection with the output of the Sigmoid function. Introducing the Sigmoid activation function to dynamically regulate the information flow in the skip connections of residual networks not only increases the model's complexity and flexibility, allowing the network to adaptively balance the information ratio between the direct path and the nonlinear path but also enhances the model's robustness to changes in input data, helping to mitigate overfitting issues. Furthermore, this mechanism, by providing additional dynamic elements, helps maintain the stability of the training process, especially reducing the risk of gradient vanishing or exploding in the training of deep networks. Experiments show that the improved residual blocks outperform traditional residual blocks in metrics.

### Attention mechanism for feature refinement

#### Channel attention

**Figure 3:**
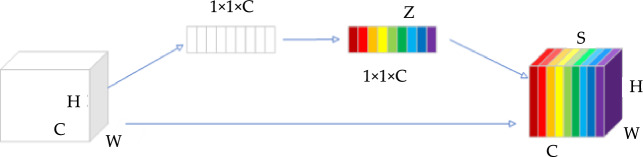
Channel attention.

While the addition of residual connections can enhance the precision in extracting water bodies, relying solely on this modification is not adequate for attaining optimal results. The challenge lies in the fact that while residual connections alleviate the problem of information loss to some extent, they do not define the features of each channel. Therefore, we have introduced a channel feature attention mechanism. The channel attention mechanism plays a crucial role in deep learning, dynamically reinforcing or suppressing the responses of various channels in a convolutional neural network. It allows the model to dynamically adapt its attention and prioritize the crucial aspects of the current task. By aggregating global information, such as through global average pooling or max pooling, this mechanism captures global statistical information to identify and emphasize important channels, thus providing an effective way to refine and enhance the model's feature representation^21^. Assuming there is a feature map $F \in R^{H \times W \times C}$ where *H*, *W* and *C* (*H* and *W* signify the feature map's height and width, respectively, and *C* stands for the particular channel index.) Initially, the spatial information is aggregated utilizing global average pooling in order to compress channels. For the c channel, its global average pooling is denoted as *Z*_*c*_, as defined in Eq. (1).

1 $$Z_{c} = \frac{1}{H \times W}\sum\limits_{i = 1}^{H} {\sum\limits_{j = 1}^{W} {F_{i,j,c} } }$$

where $\frac{1}{H\times W}$ denotes the normalization factor, used to calculate the average over the spatial dimensions. $\sum\limits_{i = 1}^{H} {\sum\limits_{j = 1}^{W} {} }$ is a double summation symbol, indicating the summation over all elements across the height and width. The pixel value of ${F}_{i,j,c}$ in the original feature map corresponds to channel c, row *i*, and column *j*. Subsequently, to produce the weights for each channel as defined in Eq. (2), a fully connected layer, a ReLu activation function, and a Sigmoid activation function are employed.

2 $$s=\sigma \left(g\left(z,W\right)\right)=\sigma \left({W}_{2}*ReLU\left({W}_{1}*z\right)\right)$$

where *s* contains the weights for each channel. The Sigmoid activation function, denoted by $\sigma$, is employed. $g(z,W)$ represents a function of a fully connected layer that takes the channel descriptor z and a learned weight matrix *W* as inputs, outputting a transformed vector. ${W}_{2}$ and ${W}_{1}$ correspond to the weight matrices of two fully connected layers, where ${W}_{1}$ acts as the dimension reduction layer while ${W}_{2}$ serves as the dimension increase layer. Ultimately, the acquired weights s are utilized on the initial feature map. The output feature map of the c-th channel after channel attention processing is defined as Eq. (3). Figure 3 shows the structure of the channel attention.

3 $${\widetilde{F}}_{c}={s}_{c}\cdot {F}_{c}$$

#### Spatial attention

**Figure 4:**
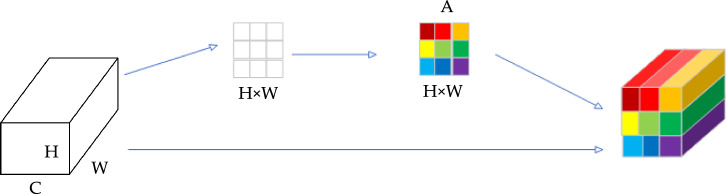
Spatial attention.

The underlying principle of the channel attention mechanism is to utilize channel weights to extract valuable information. By considering different weights, a feature map summation can be performed per this principle. This mechanism, known as spatial attention, involves incorporating weight information from various sections of the feature map. The spatial attention mechanism can identify and concentrate on the most critical parts of an image, significantly enhancing the model's efficiency and accuracy in processing visual information. This mechanism uses attention maps generated by convolutional layers to assign weights to each spatial location, thereby reinforcing the response to important features and suppressing irrelevant areas^21^. This allows the network to focus more on task-relevant signals in complex backgrounds. Assuming there is a feature map $F\in {R}^{H\times W\times C}$, the spatial attention mechanism can be defined as shown in Eq. (4).

4 $$A=\sigma \left({f}_{\text{att}}\left(F\right)\right)$$

here, *A* is the generated spatial attention map, whose dimensions are the same as the input feature map. The Sigmoid activation function, denoted as $\sigma$, is employed to introduce non-linearity into the neural network model. On the other hand, the convolution operation, represented by ${f}_{att}$, plays a crucial role in learning and adapting the weights of the feature map. Finally, through the dot product, a new weighted feature map is obtained. The new feature map obtained after spatial attention processing is defined as Eq. (5). Figure 4 shows this structure.

5 $$\widetilde{\text{F}}=\text{A}\odot \text{F}$$

#### Design of channel attention mechanism and spatial attention mechanism

The previous sections introduced channel attention and spatial attention mechanisms; this section will describe how to design this module. In the baseline U-net model, the flow of an image can be summarized as the extraction of simple to deep features of an image followed by the re-modeling of deep image features. In the final output, specifically the classification of individual pixels, accuracy essentially depends on the modeling of deep features. Under the same conditions of downsampling and upsampling, the finer the deep features, the more precise the re-modeling of deep image features, and hence, the more accurate the classification of individual pixels. Considering that high-resolution remote sensing imagery allows for clearer modeling of similar water bodies, this paper decides to integrate the channel attention mechanism after the third downsampling. This integration considers both the extraction of deep features and the selection of shallower features. Although the channel attention mechanism does not directly affect contextual information, by weighting features across different channels, the model can pay more attention to feature channels that are more important for the current task. This operation indirectly influences the extraction of contextual information.

The spatial attention mechanism, by focusing on key areas within an image, enables the network to more accurately identify water bodies and their boundaries. This mechanism enhances water body detection accuracy in complex environments by allocating higher weights to pixels related to water features while suppressing interference from the background or non-water regions. The penultimate layer of upsampling in U-net retains high-level abstract features extracted during the downsampling process and integrates shallow information through skip connections. Therefore, the penultimate layer of upsampling is rich in semantic information, prompting the decision to incorporate spatial attention mechanisms at this stage. Through this approach, the network can capture more detailed features of water bodies' boundaries and internal textures, which is particularly important for accurately depicting small water bodies or edges of water bodies in high-resolution remote sensing images.

### Multi-scale dilated convolution module for spatial contextual relationship modeling

**Figure 5:**
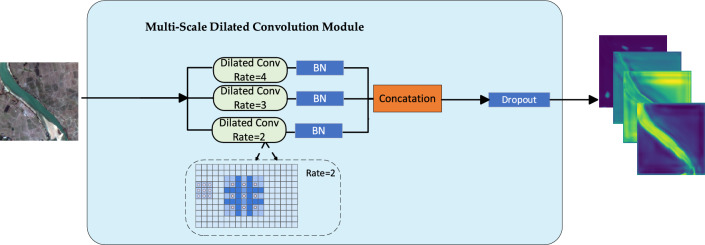
Diagram illustrating the expansion of the receptive field by dilated convolution.

Due to the structure of U-net, there is a significant loss of detail in the transfer of feature maps to the right, resulting in reduced accuracy in the recognition of small water bodies. To address this issue, this paper designs a Multi-Scale Dilated Convolution (MSDC) module containing multiple dilated convolutions with different kernel sizes^35^. Standard convolution operates on the input image via a sliding window (the convolutional kernel), where each window performs a weighted summation of the covered pixels to generate the pixel values for the output feature map. Dilated convolution introduces an additional parameter known as the dilation rate, which defines the spacing between elements within the convolutional kernel. In standard convolution, the receptive field increases linearly with network depth. Dilated convolution allows for a more rapid increase of the receptive field without a significant increase in parameters or computational load. This means that the neural network can capture a broader context at deeper levels. The MSDC module designed in this study is illustrated in Fig. 4. To enhance the receptive field, a higher dilation rate is necessary for dilated convolutions in the shallow layers of both the encoder and decoder components within the U-net architecture, particularly in regions with larger feature maps. Conversely, to ensure the preservation of intricate details and accommodate abstract feature maps, reducing the dilation rate in both the encoder and decoder modules of the U-net is advisable. This process can be simply considered as setting a smaller dilation rate, or even zero, for more abstract features. Figure 5 illustrates the use of multi-scale dilated convolution. The three branches in Fig. 5 (from top to bottom) represent the data flow of EU-Net. The left side can be understood as low-level features, with features becoming more advanced (downsampling layers) towards the right. In the shallow-level features, a larger dilation rate is required. As the data flow moves towards the deeper layers of the network, the required dilation rate gradually decreases. Then, batch normalization (BN) processing is performed, followed by concatenation to obtain the merged features. Let each branch's feature map be represented by $F_{i}$, where $i$ denotes the branch index, with $i = 1$ being the leftmost (low-level features), and as we move to the right (towards higher-level features), the value of $i$ increases. For each branch $i$, a dilation operation $D_{i}$ is applied to the feature map $F_{i}$ with a dilation rate $r_{i}$. The dilation rate $r_{i}$ is larger for lower-level features ($i$ being smaller) and decreases for higher-level features ($i$ being larger). This can be defined as Eq. (6):

6 $$F_{i}^{\prime} = D_{i} \left( {F_{i} ,\tau_{i} } \right)$$

where $F_{i}^{\prime}$ is the feature map after applying dilation, and $D_{i} ( \cdot ,r_{i} )$ represents the dilation operation on feature map $F_{i}$ with a dilation rate $r_{i}$​.

After the dilation operation, each feature map $F_{i}^{\prime}$ undergoes batch normalization (BN), denoted as $BN(F_{i}^{\prime} )$.

Finally, the processed feature maps from all branches are concatenated to form the merged feature map is definded as Eq. (7):

7 $$F_{merged} = Concat(BN(F_{1}^{\prime} ),BN(F_{2}^{\prime} ), \ldots, BN(F_{n}^{\prime} ))$$

where $Concat(\cdot)$ represents the concatenation operation over all processed feature maps from the branches, and $n$ is the total number of branches in the EU-Net architecture.

Therefore, using convolutions with different dilation rates at various sampling layers of U-net can effectively solve the problem of insufficient receptive fields. Overall, MSDC extract spatial context information over a larger receptive field while also taking into account the integration of multi-scale spatial relationships.

### Multi-scale feature fusion module

In the realm of remote sensing and particularly in water body extraction tasks, capturing and understanding the diverse range of spatial details is crucial. Traditional Convolutional Neural Networks (CNNs) often struggle to adapt to the diversity of objects in natural scenes, especially the more specific descriptions provided by high-resolution satellite imagery. Therefore, the fusion of multi-scale features becomes crucial, as this operation can distinguish more complex scenes.

Integration of features at multiple scales is not just a mere aggregation of features, it's a sophisticated strategy that intelligently combines and refines information from different layers of the network. Each layer of a CNN captures features at a different level of abstraction. The earlier layers tend to capture fine details such as edges and textures, while deeper layers capture higher-level semantic information like shapes and object categories. In the context of water body extraction, the finer details might include the ripples and edges of water bodies, while the higher-level features might represent the overall shape and extent of lakes or rivers. Through the integration of these multi-level features, the model achieves a more comprehensive perception of the image. This allows it to more precisely identify the locations and boundaries of water bodies. This is particularly beneficial in complex scenarios where the water body is surrounded by similar textures or where the water body's shape is irregular and intertwined with other land cover types.

**Figure 6:**
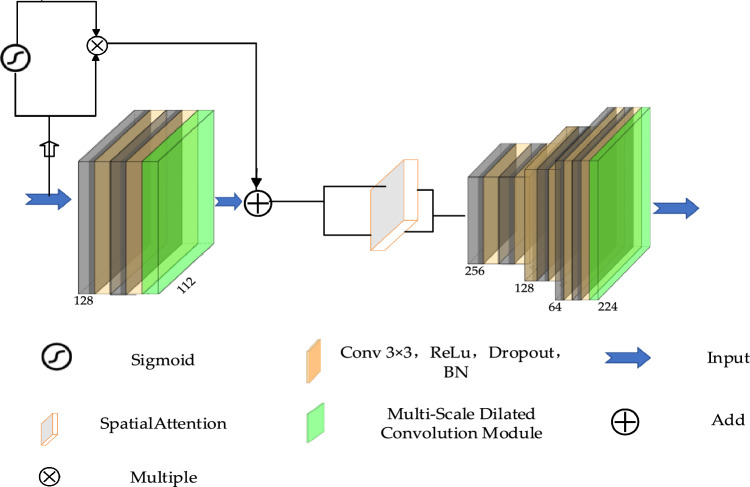
Multi-scale Feature Fusion Module.

The proposed EU-Net incorporates a multi-scale feature fusion process, where the feature maps from the last three layers of the decoder are concatenated together, as illustrated in Fig. 6. The flow of feature maps represents the complexity of feature map details. In the concatenation stage, lower-level feature maps are actually combined with higher-level feature maps. This feature map then flows into a spatial attention mechanism, enabling the network to more completely depict the details. Subsequently, the receptive field is expanded, and contextual relationships are perceived by utilizing dilated convolution on the feature map. These three types of features sequentially transition from lower-level feature maps (large water bodies) to higher-level feature maps (detailed contours). The last three layers of the decoder are rich in semantic information having gone through multiple stages of feature refinement. By fusing these layers, the model leverages both the detailed textural information and the broader contextual understanding, essential for accurately delineating water bodies of various sizes and shapes. The implications of multi-scale feature fusion are profound. For larger water bodies, which may span across several pixels and exhibit complex interactions with their surroundings, the model can utilize the broader semantic information to understand the overall context and make more informed decisions. For smaller water bodies, which might be just a few pixels wide and easily confused with other similar features, the model relies on the finer textural details to make precise localizations.

Moreover, multi-scale feature fusion also lends itself to robustness. In real-world scenarios, the conditions under which images are captured can vary widely-lighting conditions, seasons, cloud cover, etc., all introduce variability into the data. A model that relies on a single scale of features is more susceptible to being misled by these variations. In contrast, a model that fuses multiple scales of features is more resilient, drawing on a wider range of cues to make its predictions.

To summarize, the integration of multi-scale features marks a notable advancement in remote sensing and the extraction of water bodies. The proposed method acknowledges and addresses the inherent complexity and variability of natural scenes, providing a more sophisticated and comprehensive understanding of the data. By doing so, it not only enhances the performance of models on current tasks but also opens up new avenues for future exploration and innovation.

## Experimental results and analysis

To confirm the efficacy of the proposed EU-Net model, this research carried out a range of comparative experiments and ablation studies in relation to current models and methods. The dataset used is derived from the publicly available and widely used GID dataset^36^ and WHDLD dataset^37^. The GID dataset includes 150 high-definition images from over 60 regions across China, and due to its extensive coverage, the GID dataset represents a diverse geographical distribution. In data preprocessing, this paper creates a new dataset called GID-Water based on GID dataset, which ensures a better balance of positive and negative samples. The WHDLD dataset consists of images cropped from large RS (remote sensing) images of the Wuhan city area. In the WHDLD dataset, the pixels in each image are manually labeled into six categories: buildings, roads, pavements, vegetation, bare soil, and water^37^. In this part, comparisons will be made with other classic deep learning algorithms.

### Data source and processing

The GID dataset comprises high-resolution remote sensing images, assembled from Gaofen-2 satellite imagery. The Gaofen-2 satellite is the second addition to the High-definition Earth Observation System series, launched by the China National Space Administration (CNSA). Equipped with two Panchromatic and Multispectral (PMS) sensors, this satellite provides a spatial resolution of 1 m for panchromatic imagery and 4 m for multispectral imagery. It covers a combined swath width of 45 km. The dataset obtained from this satellite has a sub-satellite point resolution of 0.8 m for panchromatic images and 3.24 m for multispectral images. Each camera offers a viewing angle of 2.1 degrees, and annotations are available at the pixel level for a total of 150 images in the dataset. The GID dataset includes multispectral images capturing wavelengths in blue (0.45–0.52 µm), green (0.52–0.59 µm), red (0.63–0.69 µm), and near-infrared (0.77–0.89 µm) bands, with each image having dimensions of 6800 * 7200 pixels.

**Figure 7:**
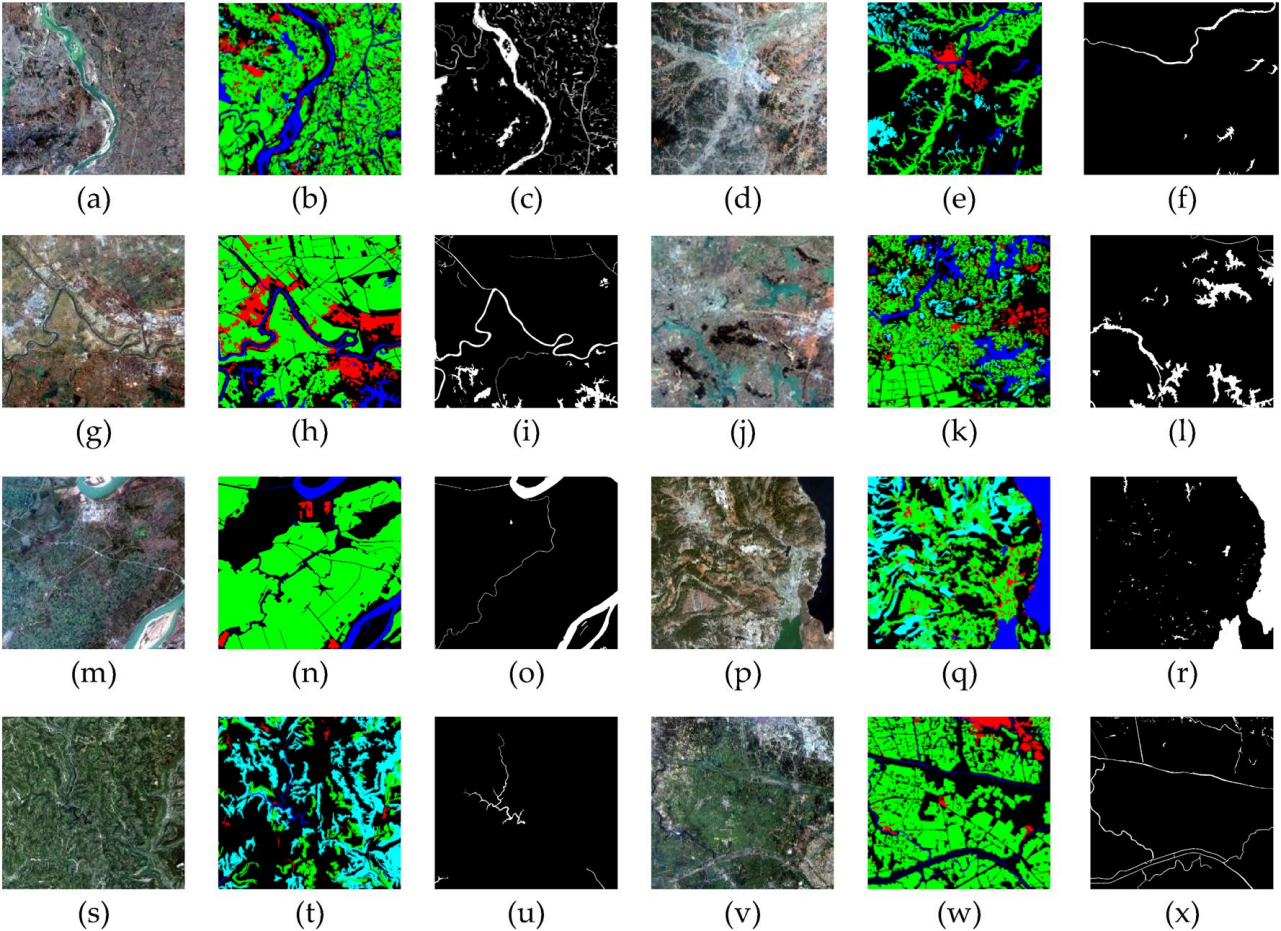
Part of GID dataset images and their corresponding labels^38^.

The GID dataset includes a vast amount of data with complex textures. From the label annotations as shown in Fig. 7, it is divided into six categories: red for buildings, green for farmland, light blue for forests, yellow for grassland, dark blue for water bodies, and black for unknown classification. Although the GID dataset provides abundant data for multi-task classification, it has shortcomings in water body recognition. For instance, the data size is too large, there are many non-water body images, a wide variety of label categories, and a lack of division between validation and training sets.

In response to the aforementioned drawbacks, this paper has made the following improvements:

1. Due to the high-resolution nature of the GID dataset that consumes a significant amount of VRAM when read, it cannot be directly trained in the network. Therefore, in data processing, this paper considers dividing each high-resolution image and its corresponding label into 224*224 pixel blocks, which, when pieced together, still form the original image.
2. As shown in Fig. 7c and e below, non-water body images dominate the dataset. In the segmentation of high-resolution images, the presence of water bodies typically does not dominate, resulting in a significant imbalance between positive and negative samples. This imbalance could hinder the model's accurate detection of positive samples. To address this issue, our study proposes an innovative integrated dataset called GID-Water, designed to exclude blocks that do not contain water bodies. This approach ensures a more balanced distribution of positive and negative samples.
3. Due to the intricate nature of labeling for water body extraction tasks in training networks, we have simplified the previously mentioned six categories into two distinct ones: water bodies and non-water bodies. To accomplish this, we substituted the initial dark blue annotation denoting water bodies with white and black annotations representing non-water bodies, as depicted in Fig. 7a–c.
4. The prepared dataset is split into training and validation subsets, maintaining a proportion of 80% for training and 20% for validation.

**Figure 8:**
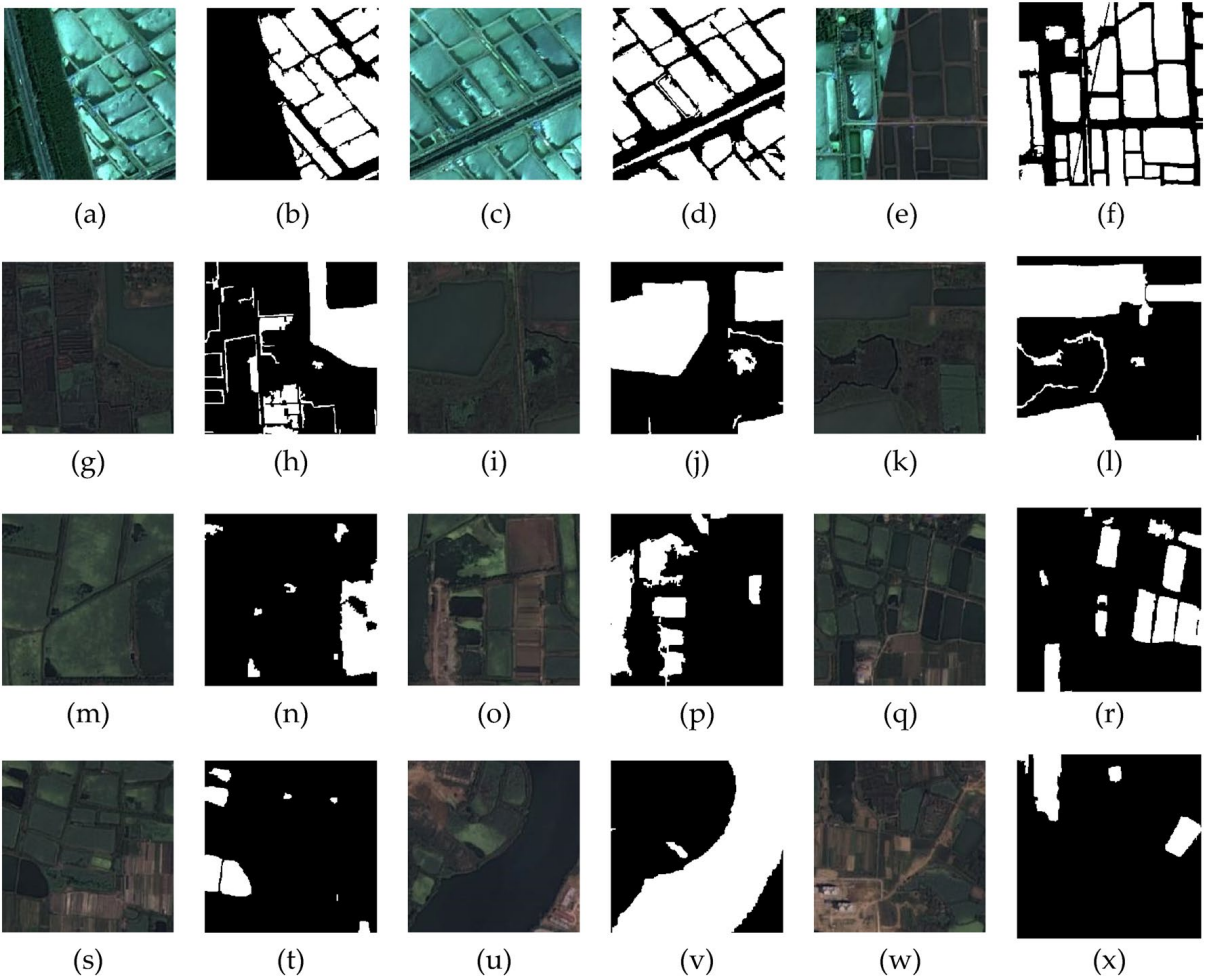
Part of WHDLD dataset images and their corresponding labels.

The WHDLD dataset has a resolution of 2 m, and Fig. 8 represents a portion of this dataset.

### Experimental setup and accuracy evaluation indicators

During the training process, our hardware configuration consisted of an NVIDIA GeForce RTX 4090 graphics card and 64GB of RAM. The Linux operating system was used with Python version 3.8 and PyTorch version 2.1.0 for environment setup. Following each epoch, a validation procedure was conducted to evaluate metrics, including Average Accuracy, Precision, Intersection over Union (IoU), F1 Score, and Recall. Furthermore, the trained model parameters were saved at this stage.

The Average Accuracy metric measures the model's ability to accurately classify instances in both binary and multi-class scenarios. It is calculated by determining the accuracy for each class and then taking the average of these accuracies. The calculation method for average accuracy is defined by Eq. (8).

8 $$\text{Average Accuracy}=\frac{1}{N}\sum_{i=1}^{N}\frac{{\text{TP}}_{i}+{\text{TN}}_{i}}{{\text{Total Samples}}_{i}}$$

Variable $\text{T}{\text{P}}_{\text{i}}$ represents the model's count of positively predicted positive samples, while $\text{T}{\text{N}}_{\text{i}}$ denotes the count of accurately predicted negative class samples. The variable $\text{T}{\text{otal Samples}}_{i}$ refers to the overall sample size, indicating each group's total number of samples. The calculation method of precision is defined by Eq. (9).

9 $${\text{Precision}}=\frac{\text{TP}}{{\text{TP}}+{\text{F}}{\text{P}}}$$

where $\text{F}{\text{P}}$ represents the count of misclassified negative class samples as positive by the model, IoU quantifies the overlap between the predicted positive region and the ground truth positive region in image segmentation. It is calculated by the ratio of their intersection's area to their union's area. This is an essential metric for assessing segmentation tasks. The specific algorithm of IoU is defined by Eq. (10).

10 $${\text{IoU}}=\frac{\text{TP}}{{\text{TP}}+{\text{FP}}+{\text{FN}}}$$

Recall, also known as the Sensitivity or True Positive Rate, represents the proportion of correctly identified positive samples by the model among all existing positive samples. A higher recall indicates a reduced occurrence of false negatives (missed positive samples).The specific calculation method of Recall is defined by Eq. (11).

11 $${\text{Recall}}=\frac{\text{TP}}{{\text{TP}}+{\text{FN}}}$$

The F1 Score is a performance metric combining Precision and Recall using the harmonic mean, assigning equal importance to both aspects. Its specific calculation method is defined by Eq. (12).

12 $$\text{F1 Score}=2\cdot \frac{{\text{Precision}}\cdot {\text{Recall}}}{{\text{Pr}}{\text{ecision}}+{\text{Recall}}}$$

### Comparison of water extraction accuracy with existing models

To verify the effectiveness and superiority of the proposed EU-Net, DeepLabV3+^25^, FCN^23^, SegNet^39^, UNet++^22^, U2-Net^40^, UNeXt^33^, PSPNet^41^, Dfanet^42^ CMLFormer^43^, CMTFNet^44^ and ViT^28^,were used for comparison experiments. The Table 1 clearly shows the performance differences among different network architectures in the task of water body extraction. U-net, as a baseline model, exhibits relatively lower performance in terms of accuracy, IoU, F1 score, and recall rate. In contrast, EU-Net with enhanced features significantly improves model performance, showing notable improvements in all performance metrics-an average accuracy of 0.9731, IoU increased to 0.9351, Recall and F1 score rate reaching 0.9649 and 0.9662 respectively. Although the metrics of DeepLabV3+ are close to those of EU-Net, it does not perform at a similar level as EU-Net in the actual task of waterbody segmentation in remote sensing. FCN performs slightly below EU-Net and DeepLabV3+ but still surpasses the baseline U-net model. SegNet shows good performance in terms of accuracy and recall rate, validating its utility in specific scenarios. UNet++, U2-Net, and UNeXt, although having similar performance indicators as EU-Net, still do not achieve the desired water body extraction effects under complex conditions. Table 1 shows that EU-Net is still dominant when compared with the model combined with Transformer.Thus, EU-Net exhibits the best performance in the current water body detection task, while PSPNet, Dfanet, FCN, and SegNet prove to be practical, lightweight choices for applications with limited resources.

**Table 1:** Comparison of accuracy with other networks on GID dataset.

Name	Average accuracy	Precision	IoU	F1 Score	Recall
**EU-Net**	**0.9731**	**0.9676**	**0.9351**	**0.9662**	**0.9649**
CMLFormer	0.9593	0.9515	0.9033	0.9487	0.9459
CMTFNet	0.9557	0.9431	0.8964	0.9448	0.9465
UNeXt	0.9703	0.9655	0.9286	0.9627	0.9599
ViT	0.9594	0.9526	0.9034	0.9487	0.9450
DeepLabV3+	0.9721	0.9695	0.9325	0.9648	0.9604
U2-Net	0.9681	0.9607	0.9237	0.9600	0.9593
UNet++	0.9683	0.9645	0.9238	0.9600	0.9558
FCN	0.9493	0.9329	0.8831	0.9372	0.9418
SegNet	0.9072	0.8742	0.8051	0.8904	0.9154
Dfanet	0.9515	0.9455	0.8853	0.9384	0.9319
PSPNet	0.9598	0.9551	0.9041	0.9491	0.9435
U-net	0.7239	0.3702	0.3619	0.4199	0.5000

Significant values are in bold.

**Figure 9:**
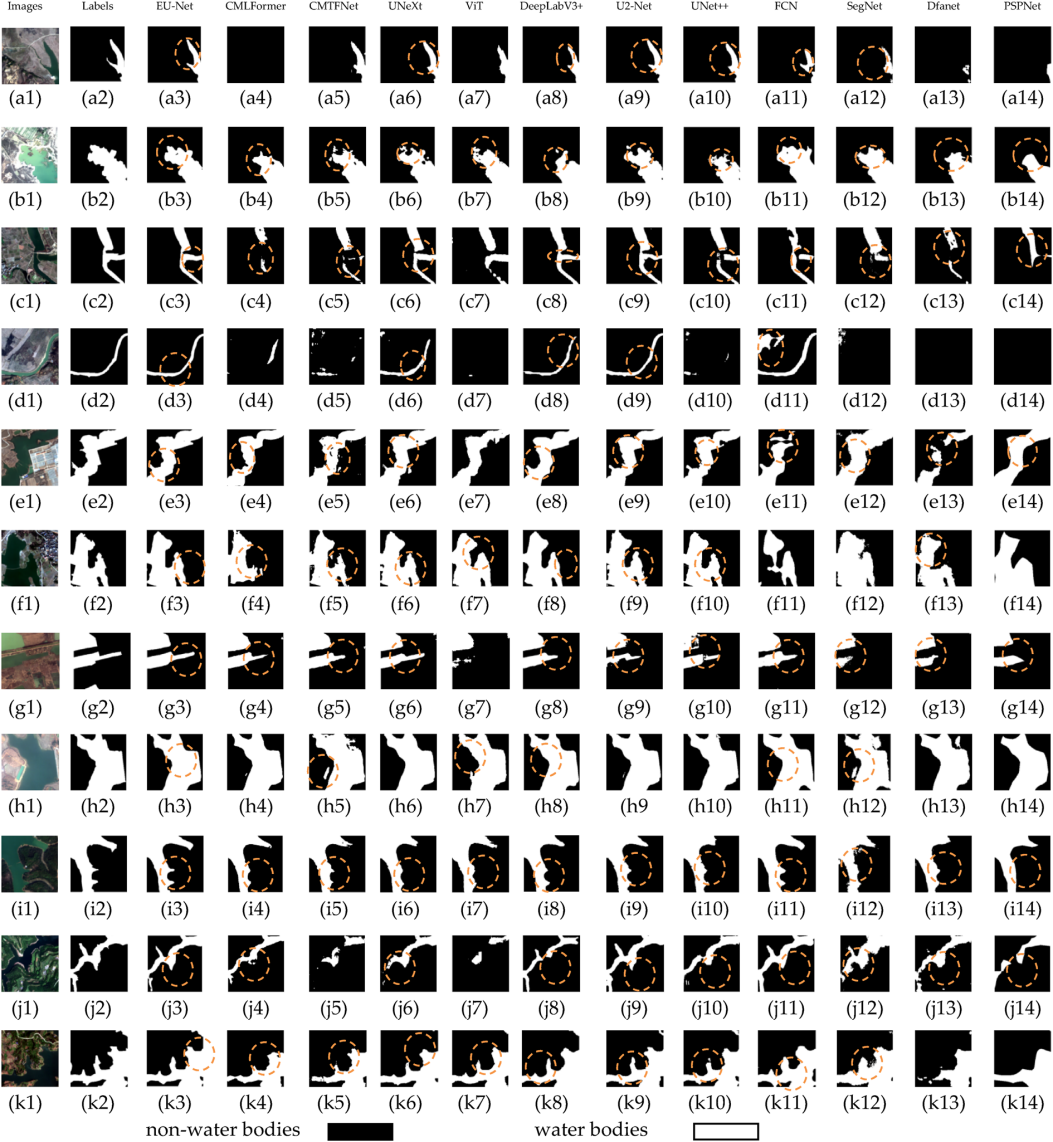
The performance evaluation of various semantic segmentation models, including EU-Net, CMLFormer, CMTFNet, UNeXt, ViT, DeepLabV3+, U2-Net, UNet ++, FCN, SegNet, Dfanet, PSPNet,was conducted on the GID-Water dataset. The original images (**a1**) to (**k1**) were utilized while corresponding labels (**a2**) to (**k2**) indicated non-water bodies in black areas and water bodies in white areas^38^.

This sub-section describes a comparative analysis of water body segmentation results from different networks. Figure 9 displayed these results. In the first row of Fig. 9 (a1), EU-Net and DeepLabV3+ capture more detailed water body areas, closely resembling the actual labels (a2), whereas FCN and SegNet miss significant areas. In UNet++, U2-Net, and UNeXt networks, there is a loss of classification for edges and small water bodies, while Dfanet and PSPNet show extensive distortion. In (b1), EU-Net remains the strongest in boundary classification, with the other networks exhibiting significant distortion. As the image complexity increases, rows (c) and (d) display the models' differing performances in handling complex backgrounds and adjacent non-water areas. Particularly in Fig. 9 (d1), only EU-Net and DeepLabV3+ successfully avoid mislabeling Dfanet green vegetation as water. In scenarios where artificial structures meet water bodies, as shown in Fig. 9 (e1), FCN and SegNet tend to wrongly classify these structures as water, UNet++, U2-Net, UNeXt misjudge more water as non-water, and and PSPNet have severe distortion, while EU-Net and DeepLabV3 + provide more accurate segmentation, but DeepLabV3+ is not as good as EU-Net in boundary handling. In Fig. 9 (f1), while all models perform well in segmenting larger water bodies, EU-Net shows more accuracy in continuity and completeness when segmenting smaller water bodies. Overall, EU-Net excels in maintaining water body geometry and boundary clarity and is less prone to under-segmentation or over-segmentation due to environmental influences.

Further predictions showcase each network's performance in different types of water body scenes. In Fig. 9 (g1), almost all models recognize large water bodies, but apart from DeepLabV3+, other networks suffer from severe boundary information distortion, whereas EU-Net shows segmentation advantages in identifying small water bodies and narrow channels. In Fig. 9 (h1) and (i1), EU-Net's ability to maintain complex water body shapes and boundary integrity is observed. In the Fig. 9 (j1), all models distinguish the main water body, but EU-Net avoids over-segmentation issues, providing smoother and more accurate edges. As scene complexity increases, as shown in the Fig. 9 (k1), EU-Net continues to accurately segment narrow and small waterways, while other networks are insufficient in these areas. These results indicate that while all models can segment large-scale water bodies, EU-Net possesses higher precision and robustness in identifying small-scale or geometrically complex water bodies. Overall, EU-Net and DeepLabV3+ perform well in water body segmentation tasks, especially in capturing details and maintaining segmentation quality.

To further demonstrate the superior performance of the EU-Net model in water body extraction, this paper also tested it using WHDLD dataset. The WHDLD dataset is a dense labeled RS dataset, which can be applied to water extraction tasks^45^.The results, shown in Table 2, indicate that EU-Net's metrics still rank first, with the DeepLabV3+ model slightly behind EU-Net. The performance metrics of the other models do not significantly differ from those in Table 1. Therefore, the EU-Net model exhibits the best performance in the task of water body extraction.

**Table 2:** Comparison of accuracy on WHDLD dataset.

Name	Average accuracy	Precision	IoU	F1 score	Recall
**EU-Net**	**0.9721**	**0.9714**	**0.9256**	**0.9653**	**0.9613**
CMLFormer	0.9622	0.9673	0.9011	0.9472	0.9306
CMTFNet	0.9640	0.9588	0.9075	0.9509	0.9435
UNeXt	0.9624	0.9674	0.9018	0.9476	0.9312
ViT	0.9607	0.9597	0.8985	0.9458	0.9336
DeepLabV3+	0.9715	0.9662	0.9314	0.9642	0.9622
U2-Net	0.9658	0.9671	0.9109	0.9528	0.9403
UNet++	0.9529	0.9567	0.8780	0.9338	0.9155
FCN	0.9694	0.9633	0.9266	0.9616	0.9599
SegNet	0.9386	0.9415	0.8432	0.9128	0.8912
Dfanet	0.9048	0.9206	0.7582	0.8564	0.8207
PSPNet	0.9422	0.9458	0.8518	0.9181	0.8970
U-net	0.7501	0.5098	0.3773	0.4334	0.5002

Significant values are in bold.

### Ablation study

**Figure 10:**
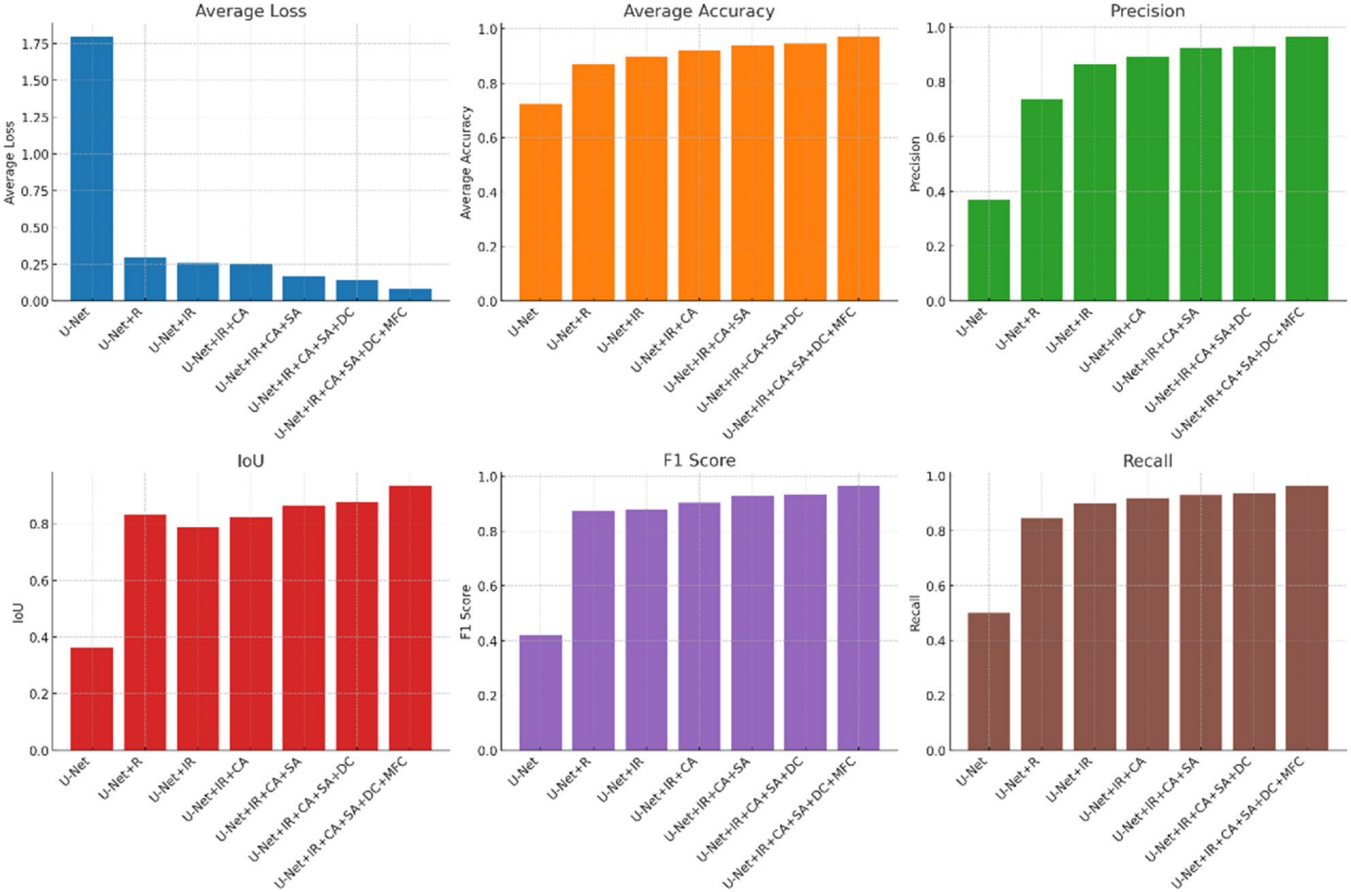
Ablation experiments on GID dataset. R stands for residual block, IR stands for improved residual block, CA stands for channel attention, SA stands for spatial attention, DC stands for dilated convolution, and MFC stands for multi-scale feature fusion.

**Figure 11:**
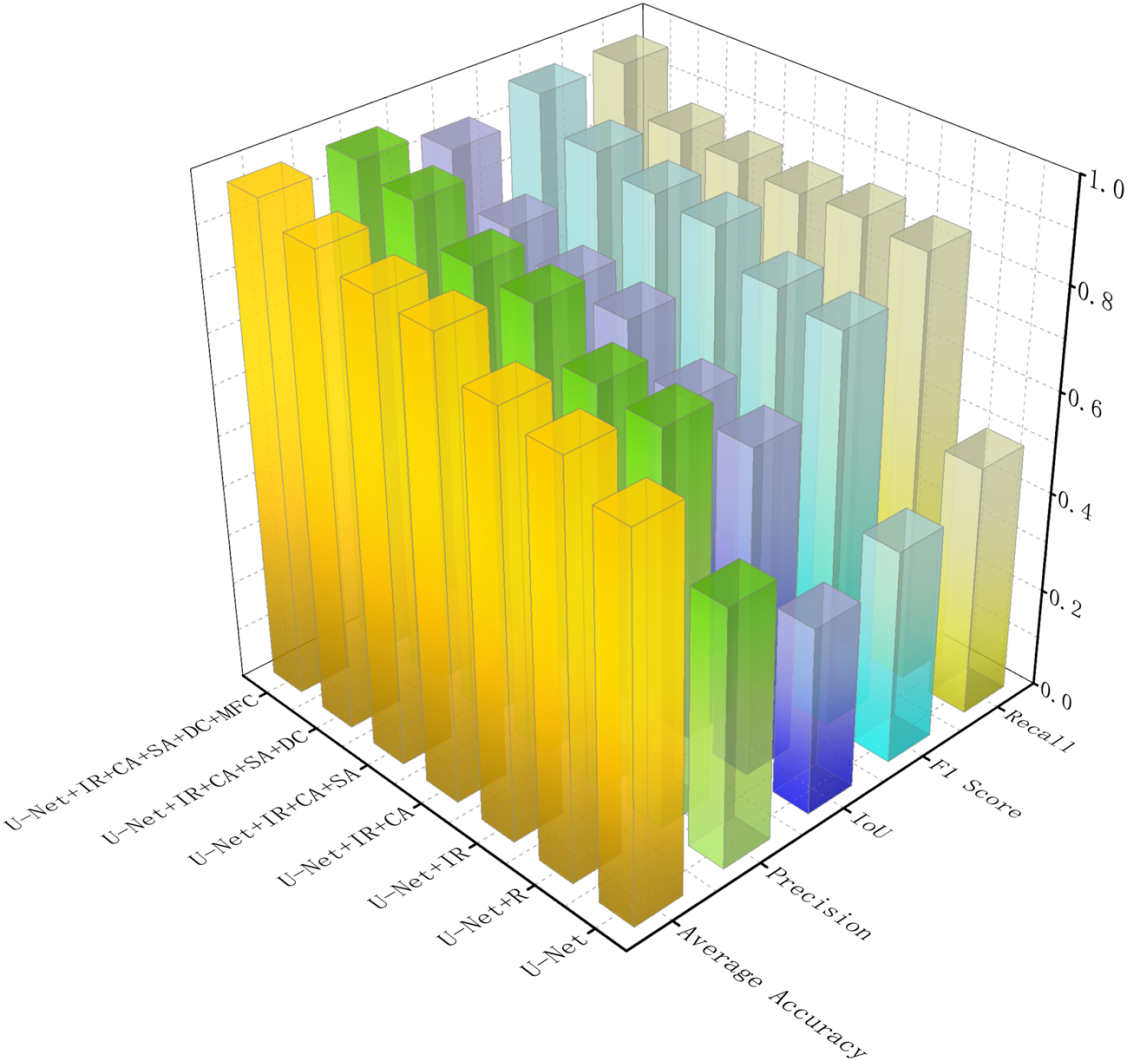
Ablation experiments on WHDLD dataset. R stands for residual block, IR stands for improved residual block, CA stands for channel attention, SA stands for spatial attention, DC stands for dilated convolution, and MFC stands for multi-scale feature fusion.

**Figure 12:**
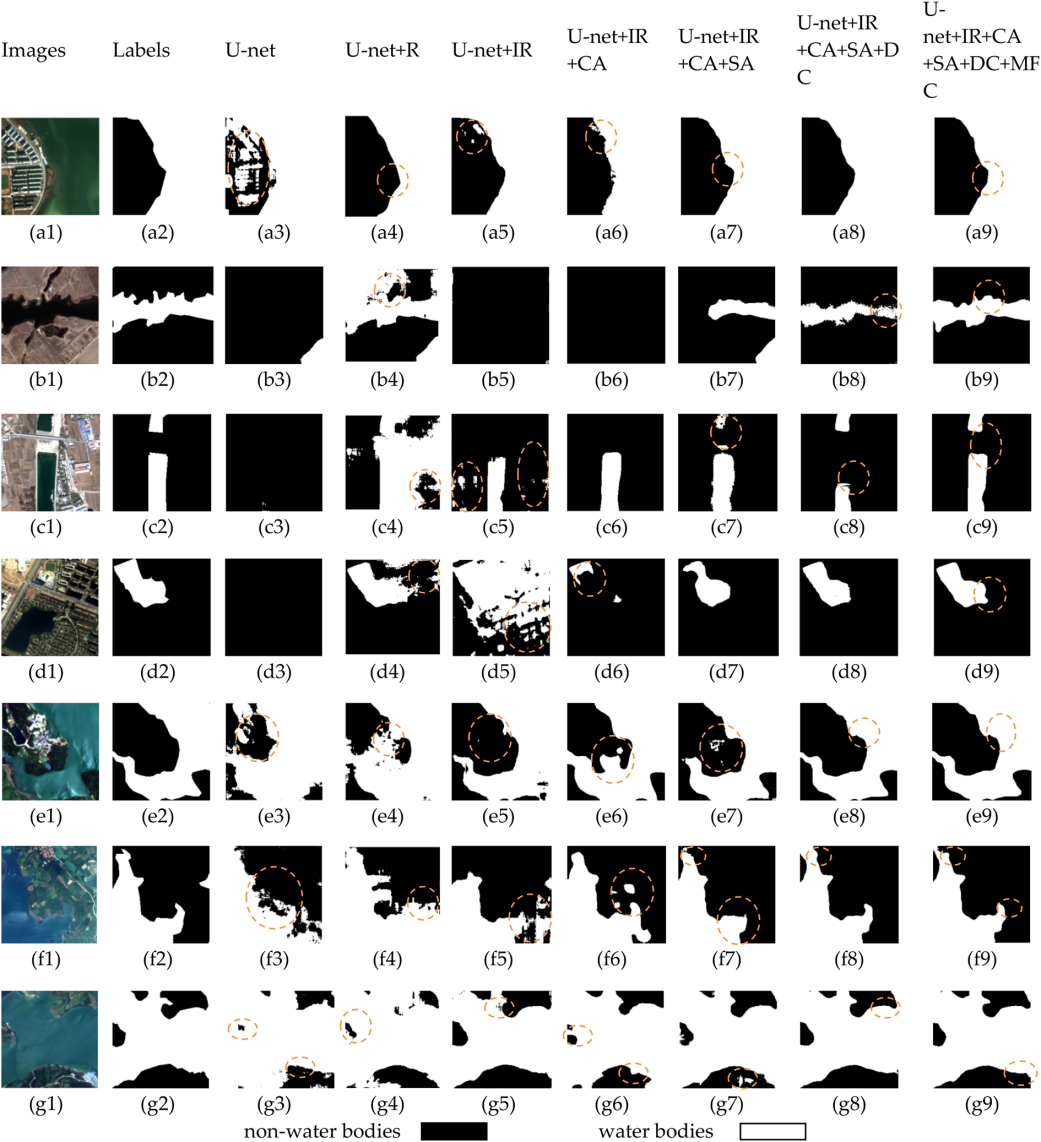
Comparing U-net with different modules added, where (**a1**) to (**k1**) are the original images, (**a2**) to (**k2**) are the labels, with black parts indicating non-water bodies and white parts indicating water bodies^38^.

In our research, through ablation studies, we incrementally introduced different model components to assess their individual impact on performance. To clearly display the accuracy of the experiments, we summarized each trial in tables. Figure 10 shows the results of the networks tested on the GID dataset, and Fig. 11 displays the outcomes of the networks on the WHDLD dataset. To visually demonstrate the differences, we also compiled predicted images, with the results shown in Fig. 12. In water body segmentation, a challenging category of images is those that resemble water bodies but are not, which we refer to as confounding images, such as in Fig. 12 (b1).

The baseline U-net model demonstrated preliminary segmentation capabilities, with a high average loss indicated in the table, suggesting room for improvement. From the prediction Fig. 12 (b3), (c3), (d3), it is evident that U-net lost considerable semantic information in confounding images and was nearly incapable of correctly classifying complex water bodies. With the addition of residual blocks (R), we observed a significant reduction in average loss and improvements in accuracy, precision, IoU, and F1 score, indicating that residual connections aid in capturing deep features and enhance the model's learning ability. As shown in Fig. 12 (a4), the introduction of residual blocks alone did not yield ideal water extraction effects, but this network began to form a basic concept of contours. When we incorporate the improved residual blocks, as seen in Figs. 10 and 11, most of the network's metrics improve. Combined with the fourth column of Fig. 12, it is evident that the network's ability to predict boundaries is significantly enhanced. This demonstrates the effectiveness of the improved residual blocks. Further, by incorporating channel attention mechanisms (CA) and spatial attention mechanisms (SA), our U-net + IR + CA and U-net + IR + CA + SA models improved on all performance metrics, suggesting that attention mechanisms further enhance segmentation precision by intensifying the model's focus on key features. U-net + IR + CA + SA performed well in classifying Fig. 12 (e1) and achieved certain segmentation accuracy for complex textured water bodies Fig. 12 (c7) and confounding Fig. 12 (b1).Finally, after introducing dilated convolution and multi-scale feature fusion modules, the model achieved optimal performance, especially evident in the improvements in IoU and F1 score, suggesting that by integrating features of various scales, the model can better handle water bodies of all sizes, which is crucial for improving model performance. From the prediction images, it is evident that the final network finely classifies confounding Fig. 12 (b1) and performs excellently across all prediction images, particularly when dealing with water bodies in areas with complex terrain and blurred boundaries, where EU-Net showed better adaptability and robustness. For instance, in areas with highlights and shadows in the images, EU-Net accurately differentiated between water and non-water regions. These results confirm that each on model performance, particularly in enhancing the model's ability to capture details.

## Discussion

With the continuous advancement of satellite technology, there is an increasing interest in utilizing high-resolution remote sensing imagery for water body extraction. The complexity of water bodies in many aspects such as shape and size makes the water body extraction work challenging^46^. For instance, high-altitude reflections and confusing ground objects can easily lead to misjudgment of water bodies. Beginning in 2014, deep learning has progressively emerged as a leading approach for object classification in remote sensing imagery. Hence, there exists substantial potential for advancements in employing deep learning methodologies to extract water from high-resolution remote sensing imagery. This paper introduces the EU-Net, a model developed on the foundation of the U-net model. Through five performance indicators and actual prediction displays, EU-Net demonstrates a good ability to extract water bodies and performs better than other networks in complex texture water body extraction.

In water extraction, different models have varying accuracies, which may be closely related to their working methods. CMLFormer and CMTFNet are recent models. CMLFormer is a cross-modal learning transformer model that introduces multi-modal interaction layers in the model to achieve effective fusion and mutual enhancement of information from different modalities. CMTFNet adopts a multi-task learning architecture, sharing feature representations among different tasks to improve the efficiency and generalization ability of the model. ViT, on the other hand, uses only the Transformer architecture for semantic segmentation. DeepLabV3+ represents the latest and most sophisticated iteration in the DeepLab series, renowned for its effective semantic segmentation capabilities in intricate scenarios. This version introduces a decoding module designed to enhance the quality of segmentation outcomes, particularly around the edges of objects^47^. Its encoder-decoder structure effectively captures richer contextual information across multiple scales and provides clearer, more precise segmentation boundaries. The method has gained a reputation for effectively achieving a balance between speed and accuracy, rendering it suitable for practical applications that necessitate precise segmentation. The pioneering application of deep learning to semantic segmentation was accomplished by introducing Fully Convolutional Networks (FCN). A vital aspect of this approach involves transforming the fully connected layers commonly found in traditional convolutional neural networks into convolutional layers. This modification empowers the network to handle input images of any size and generate segmentation maps with corresponding dimensions. FCNs upsamples the low-resolution feature maps through transposed convolution (sometimes also called deconvolution) layers, restoring the original resolution of the image. This method allows the network to learn rich feature representations and perform end-to-end training and prediction. FCNs excels in handling objects of different sizes and shapes, but its segmentation precision on details may not be as good as some later models. SegNet is a lightweight convolutional neural network designed for scene understanding. Its main feature is its encoder-decoder architecture. The encoder part adopts a structure similar to VGG16 but removes the fully connected layers, allowing it to accept input images of any size. The decoder part gradually restores the feature map size of the input image^48^. A key innovation of SegNet is the use of max-pooling indices for upsampling in the decoder, which helps preserve boundary information and reduce the number of parameters. Although SegNet performs well in handling complex scenes and defining boundaries, it may not be as precise as other more complex models. U-net is known for its excellent performance and efficient data utilization. The U-net architecture is designed with the letter "U," consisting of contracting and expanding pathways. In U-net, the contracting path functions akin to a typical convolutional network, extracting image features through successive convolution and pooling processes. Concurrently, the expanding path incrementally reconstructs the image dimensions using upsampling and convolution operations, and it merges the relevant feature maps from the contracting path. This design allows U-net to achieve good segmentation results even with a small amount of labeled data^49^.

Revealing water bodies from high-resolution remote sensing imagery encounters various challenges and obstacles^50^. Firstly, water body boundaries are complex in these images, making precise segmentation difficult, especially when vegetation, shadows, or other surface objects surround the boundaries^51^. Additionally, the spectral resemblance between water and non-water regions, like wetlands and shadows, can lead to misclassifications by models. Furthermore, the varied nature of water bodies and their changes under diverse environmental conditions further complicate the task of water identification. High-resolution images can display small-scale water bodies, but detecting these small water bodies requires a more sensitive network for recognition. The influence of lighting and shadows cannot be overlooked either, as they can alter the spectral characteristics of water areas, making water body identification more challenging. Finally, the substantial data processing capacity required to handle large volumes of high-resolution images poses higher demands on storage space and computational resources. These challenges necessitate the adoption of more advanced technologies and methodologies to achieve accurate and efficient water body extraction^37^.

In the experiment, two datasets were used. The first dataset is the GID-Water dataset, which contains a training set of over 40,000 images sized 224 * 224 with labels, and a validation set containing over 10,000 images of the same size and labels. The second dataset is named WHDLD, consisting of a training set with 4000 images sized 256 * 256 with labels and a validation set containing 1000 images of the same size and labels. The experiments were conducted on an RTX 4090 graphics card. When training on the GID-Water dataset with a batch size of 64 and for 50 epochs, the baseline model U-net required 4 h of training time. Under the same conditions, EU-Net took 5.2 h to train. DeepLabV3+ needed 4.7 h of training time. The training processes for U2-Net, UNet++, and UNext were between 4 to 5 h. The model training combined with Transformer is often significantly slower than that of EU-Net, CMLFormer,CMTFNet and ViT in more than five hours. Under the same conditions, the training costs for lightweight networks like PSPNet, Dfanet, FCN, SegNet were all within 4 h. When training on the WHDLD dataset, the duration for all networks did not exceed 3 h. Although the computational cost of EU-Net is slightly higher, it is the best choice in terms of accuracy and water body segmentation instances.

## Conclusions

Revealing water bodies in high-resolution remote sensing images is a complex task that requires precise identification. This research investigates the effective utilization of deep learning techniques to enhance the accuracy of extracting water bodies from high-resolution remote sensing images. Specifically, we propose EU-Net, an enhanced version of the U-net model called Enhanced U-net, specifically designed to optimize GF-2 high-resolution satellite imagery.

In EU-Net, a improved residual structure is introduced to enhance the model's contextual connections. A multi-scale dilated convolution module is developed, allowing the network to acquire varied receptive fields in both the encoder and decoder. Enhancing the network's capacity facilitates the analysis of interconnections among physical characteristics, thereby enabling a more efficient identification of water bodies in complex terrains. A multi-scale feature fusion module is implemented by concatenating features from different levels, transitioning from low-level to high-level features. Additionally, channel attention and spatial attention mechanisms are employed to more effectively integrate contextual relationships. Regarding the dataset, a more streamlined water body recognition dataset, GID-water, is proposed based on the GID dataset. To effectively demonstrate the excellent water body segmentation capability of EU-Net, this paper also conducted experiments on the WHDLD dataset.The EU-Net model was compared against nine widely used CNN models for semantic segmentation in computer vision. Through a quantitative analysis based on five evaluation metrics and an intuitive visual comparison, we comprehensively assessed the performance of each model in extracting surface water bodies. The findings suggest that EU-Net performs superior to other networks across various evaluation metrics, mainly exhibiting notable improvements in Average Accuracy, Precision, Intersection over Union (IoU), Recall and F1 Score. Furthermore, we conducted additional investigations into the contributions of different modules within EU-Net. By comparing variants with and without modules, this study found that they are crucial for handling confusing areas and water bodies of various sizes. Furthermore, in challenging scenarios such as urban water bodies and plateau lakes, EU-Net exhibited exceptional proficiency in accurately capturing intricate characteristics of aquatic environments while effectively mitigating disturbances caused by non-aquatic elements like mountain shadows, highways, vegetation shadows, and dark lawns.

The findings of this study suggest that as the dataset is progressively enhanced and the model further refined, EU-Net is poised to become a vital tool in large-scale, high-resolution remote sensing mapping of surface water. This will offer dependable data support for the surveillance and administration of surface water resources. Our research not only presents a novel approach to water body extraction technology but also establishes a basis for subsequent investigations in related areas.

This article has incorporated tailored improvements for extracting water bodies from high-resolution remote sensing imagery, aligning with professionalism and academic rigor requirements. Despite these improvements, there are still shortcomings. For example, the study uses high-resolution remote sensing images, but in some cases, the resolution is still insufficient to capture small or narrow water bodies. The dimensions and configuration of aquatic formations may undergo modifications over time, posing challenges for consistent and accurate water body extraction. Moreover, this network still requires substantial computational resources. Future research can focus on deeper optimizations based on the limitations and challenges identified in this article.

## Data Availability

The datasets generated during the current study are available in the [Figshare] repository [10.6084/m9.figshare.25360726, 10.6084/m9.figshare.25574181].
